# Identification of New Sources of Resistance to Wheat Stem Rust in *Aegilops* spp. in the Tertiary Genepool of Wheat

**DOI:** 10.3389/fpls.2018.01719

**Published:** 2018-11-22

**Authors:** Pablo D. Olivera, Matthew N. Rouse, Yue Jin

**Affiliations:** ^1^Department of Plant Pathology, University of Minnesota, St. Paul, MN, United States; ^2^Cereal Disease Laboratory, Agricultural Research Service, United States Department of Agriculture, St. Paul, MN, United States

**Keywords:** wild wheats, disease resistance, Ug99, genetic resources, tertiary genepool

## Abstract

Recent stem rust epidemics in eastern Africa and elsewhere demonstrated that wheat stem rust is a re-emerging disease posing a threat to wheat production worldwide. The cultivated wheat gene pool has a narrow genetic base for resistance to virulent races, such as races in the Ug99 race group. Wild relatives of wheat are a tractable source of stem rust resistance genes. *Aegilops* species in the tertiary genepool have not been exploited to any great extent as a source of stem rust resistance. We evaluated 1,422 accessions of *Aegilops* spp. for resistance to three highly virulent races (TTKSK, TRTTF, and TTTTF) of *Puccinia graminis* f. sp. *tritici*. Species studied include *Ae. biuncialis, Ae*. *caudata, Ae. comosa, Ae. cylindrica, Ae. geniculata, Ae. neglecta, Ae. peregrina, Ae. triuncialis*, and *Ae. umbellulata* that do not share common genomes with cultivated wheat. High frequencies of resistance were observed as 977 (68.8%), 927 (65.2%), and 850 (59.8%) accessions exhibited low infection types to races TTKSK, TTTTF, and TRTTF, respectively. Contingency table analyses showed strong association for resistance to different races in several *Aegilop*s spp., indicating that for a given species, the resistance genes effective against multiple races. Inheritance studies in selected accessions showed that resistance to race TTKSK is simply inherited.

## Introduction

Wheat stem rust, caused by *Puccinia graminis* Pers.:Pers. f. sp. *tritici* Eriks. & E. Henn. (*Pgt*), is a devastating disease of durum wheat (*Triticum turgidum* L. ssp. *durum*) and common or bread wheat (*T. aestivum* L.). Severe epidemics have been reported in all major wheat growing areas in the world (Roelfs, 1985; Saari and Prescott, 1985). For decades, stem rust has been under effective control through the use of genetic resistance. The occurrence and spread of *Sr31*-virulence races in the Ug99 race group in East Africa and other virulent races causing epidemics and localized outbreaks in Ethiopia (Olivera et al., 2015), Europe (Bhattacharya, 2017; Olivera Firpo et al., 2017; Lewis et al., 2018) and Central Asia (Shamanin et al., 2018), indicates that the disease is re-emerging as a threat to wheat production. Races in the Ug99 group have been detected across South, East and northern Africa, and the Middle East (Pretorius et al., 2000; Singh et al., 2015; Newcomb et al., 2016), and have the potential to reach critical wheat growing regions in the world (Park et al., 2011). The Ug99 race group has been rapidly evolving, producing variants with virulence to stem rust resistance genes including *Sr24* (Jin et al., 2008), *Sr36* (Jin et al., 2009), and *SrTmp* (Newcomb et al., 2016) that are important in stem rust resistance breeding (Singh et al., 2015).

The cultivated wheat gene pool has a narrow genetic base for resistance to the contemporary virulent races, such as TTKSK (Jin and Singh, 2006; Singh et al., 2006; Newcomb et al., 2016), TRTTF (Olivera et al., 2012); and TKTTF (Olivera Firpo et al., 2017). In order to broaden the basis of stem rust resistance in wheat breeding programs, it is necessary to identify and introgress effective genes from all genepools of wheat. Wild relatives of wheat are a tractable source of stem rust resistance genes. Indeed, a number of resistance genes derived from wild relatives of wheat appeared to be more effective against the races in the Ug99 group than *Sr* genes of wheat origin (Singh et al., 2006; Jin et al., 2007). *Aegilops* is the most closely related genus to *Triticum* (Kimber and Feldman, 1987; Jiang et al., 1994) and comprises 23 species that include diploid, tetraploid, and hexaploid genomes (van Slageren, 1994). *Aegilops* species are known to be a rich source of stem rust resistance, and several stem rust resistance genes have been transferred into cultivated wheat (Friebe et al., 1996; Schneider et al., 2008; Liu et al., 2011a,b; Olson et al., 2013a,b).

Ease of hybridization and reduced linkage drag make introgression from species in the primary gene pool preferred by wheat breeders to incorporate new alleles in their breeding programs (Feuillet et al., 2008). However, species in the secondary and tertiary gene pools constitute an important reservoir of genetic variability (Qi et al., 2007). *Aegilops* species in the tertiary genepool have not been exploited to any great extent for wheat improvement, and for resistance to TTKSK and other virulent *Pgt* races in particular. The objective of this study was to evaluate a collection of nine *Aegilops* species in the tertiary gene pool of wheat for resistance to race TTKSK and other *Pgt* races.

## Materials and Methods

### Germplasm

A total of 1,422 accessions of nine *Aegilops* species (three diploid and six tetraploid) deposited at the USDA-ARS, National Small Grain Collection (NSGC), Aberdeen, ID, were evaluated in this study. Species, the number of accessions and country of origin of each *Aegilops* species are given in Table 1.

**Table 1:** Number of accessions and country of origin of *Aegilops* species used in this study.

	*geniculata*	*cylindrica*	*biuncialis*	*triuncialis*	*comosa*	*caudata*	*neglecta*	*peregrina*	*umbellulata*	TOTAL
Turkey	79	87	82	148	1	33	125	3	66	624
Greece	34	2	85	73	58	28	27	0	0	307
Macedonia	3	5	11	21	0	0	13	0	0	53
Israel	0	1	0	0	0	0	0	48	0	49
Syria	11	0	15	9	0	0	0	7	0	42
Serbia	1	9	12	6	0	0	1	0	1	30
Cyprus	6	0	13	4	0	0	0	6	0	29
Ukraine	2	12	6	0	0	0	0	0	0	20
France	10	0	0	7	0	0	1	0	0	18
Azerbaijan	0	2	4	5	0	0	3	0	1	15
Iraq	0	1	1	4	0	1	7	0	1	15
Iran	0	0	0	10	0	0	1	0	2	13
Montenegro	4	0	0	1	0	0	7	0	0	12
Afghanistan	0	2	0	8	0	0	0	0	0	10
Others	15	24	4	9	0	1	9	5	0	67
Unknown	18	6	29	48	1	2	8	4	2	118
TOTAL	183	151	262	353	60	65	202	73	73	1422


### Inoculation, Incubation, and Disease Assessment

With the objective of identifying multiple and diverse resistance genes in individual accessions, we evaluated this *Aegilops* collection against multiple races with different virulence spectrum and origin. All accessions were characterized for reaction to three virulent *Pgt* races: TTKSK (Kenya), TRTTF (Yemen), and TTTTF (United States). Accessions resistant to the three races were further evaluated for their reaction to four additional US races (TPMKC, RKRQC, QTHJC, and QFCSC). The race designations are based on the letter code nomenclature system (Roelfs and Martens, 1988; Roelfs et al., 1993; Jin et al., 2008). Avirulence/virulence profile of the *Pgt* isolates used in the disease assessments is summarized in Table 2. Disease evaluations were conducted in two independent experiments. In each experiment, five seedlings per accession were inoculated with each race on fully expanded primary leaves 8–9 days after planting. Details on inoculation procedures and disease assessment were described by Jin et al. (2007). Disease reactions were classified according to Stakman et al. (1962). Infection types (ITs) 0, 1, and 2 were considered as resistant reactions and ITs 3 and 4 were considered as susceptible. Wheat cultivar McNair 701 (Cltr 15288) was included as susceptible check. Analyses of association via contingency tables were conducted to assess potential relationships of resistance to different *Pgt* races.

**Table 2:** Isolate designation, origin, and virulence phenotype of *Puccinia graminis* f. sp. *tritici* races used to evaluate resistance in *Aegilops* spp.

Race	Isolate	Origin	Virulence / avirulence formula
TTKSK^1^	04KEN156/04	Kenya	*Sr5 6 7b 8a 9a 9b 9d 9e 9g 10 11 17 21 30 31 38 McN* / *Sr24 36 Tmp*
TRTTF	06YEM34-1	Yemen	*Sr5 6 7b 9a 9b 9d 9e 9g 10 11 17 21 30 36 38 McN Tmp* / *Sr8a 24 31*
TTTTF	01MN84A-1-2	United States	*Sr5 6 7b 8a 9a 9b 9d 9e 9g 10 11 17 21 30 36 38 McN Tmp* / *Sr24 31*
TPMKC	74MN1409	United States	*Sr5 7b 8a 9d 9e 9g 10 11 17 21 36 McN Tmp / Sr6 9a 9b 24 30 31 38*
RKRQC	99KS76A-1	United States	*Sr5 6 7b 8a 9a 9b 9d 9g 17 21 36 McN* / *9e 10 11 24 30 31 38 Tmp*
QTHJC	75ND717C	United States	*Sr5 6 8a 9b 9d 9g 10 11 17 21 McN* / *7b 9a 9e 24 30 31 38 36 Tmp*
QFCSC	06ND76C	United States	*Sr 5 8a 9a 9d 9g 10 17 21 McN / Sr6 7b 9b 9e 11 24 30 31 36 38 Tmp*


*^*1*^Race nomenclature was based on Roelfs and Martens (1988) and Jin et al. (2008).*

### Inheritance Study

Bi-parental crosses between selected resistant accessions and a susceptible accession in five *Aegilops* species were made and F_2_ progeny were produced by selfing F_1_ plants. Seventeen F_2_ populations (four from *Ae. cylindrica*, four from *Ae. peregrina*, six from *Ae. triuncialis*, two from *Ae. umbellulata*, and one from *Ae. comosa*) were evaluated for reaction to race TTKSK to determine the inheritance of resistance based on phenotypic ratios. Chi-square (χ^2^) test was used to determine the goodness of fit to expected genetic ratios in the F_2_ generation.

## Results

A wide array of infection types was observed across the *Aegilops* spp. and ranged from highly resistant (IT 0) to highly susceptible (ITs 3+ and 4). Low ITs (; or ;1-) were frequently observed in *Ae. caudata, Ae. cylindrica, Ae. neglecta, Ae. peregrina* and *Ae. triuncialis*, whereas ITs 2- and 2-; were predominant in *Ae. biuncialis* and *Ae. geniculata*. We observed a high percentage of resistance in this *Aegilops* collection as 977 (68.8%), 927 (65.2%), and 850 (59.8%) accessions produced low infection types to races TTKSK, TTTTF, and TRTTF, respectively (Table 3). Five hundred and fifty one (38.8%) accessions were resistant to the three races evaluated. The frequencies of accessions resistant to race TTKSK varied among the species: over 80% in six *Aegilops* species (*Ae. caudata, Ae. cylindrica, Ae. geniculata, Ae. neglecta, Ae. peregrina*, and *Ae. triuncialis*), and below 30% in three species (*Ae. biuncialis, Ae. comosa*, and *Ae. umbellulata*) (Table 3).

**Table 3:** Number and frequency of *Aegilops* accessions resistant to *Puccinia graminis* f. sp. *tritici* races TTKSK, TRTTF, and TTTTF at the seedling stage.

Species	Genome	Accessions evaluated	TTKSK		TRTTF		TTTTF		Resistant to 3 races	
						
			Number	Frequency	Number	Frequency	Number	Frequency	Number	Frequency
*Ae. biuncialis*	UUMM	262	75	0.27	179	0.68	82	0.31	34	0.13
*Ae. caudata*	CC	65	54	0.83	40	0.62	50	0.77	32	0.49
*Ae. comosa*	MM	60	10	0.17	10	0.17	11	0.19	3	0.05
*Ae. cylindrica*	DDCC	151	133	0.88	1	0.01	102	0.68	1	0.01
*Ae. geniculata*	UUMM	183	145	0.80	159	0.87	156	0.86	136	0.75
*Ae. neglecta*	UUMM	202	189	0.94	183	0.91	170	0.84	158	0.78
*Ae. peregrina*	SSUU	73	64	0.88	47	0.64	24	0.33	14	0.19
*Ae. triuncialis*	UUCC	353	290	0.82	198	0.56	315	0.98	166	0.47
*Ae. umbellulata*	UU	73	17	0.23	33	0.45	17	0.23	7	0.10
TOTAL		1,422	977	0.69	850	0.60	927	0.65	551	0.39


Pairwise association for resistance to races TTKSK, TRTTF, and TTTTF exhibited variation among species and pathogen races. Over 75% of the accessions of *Ae. geniculata* and *Ae. neglecta* were resistant to races TTKSK, TRTTF, and TTTTF (Table 3). Resistance to pairs of the three *Pgt* races in *Ae. geniculata* and *Ae. neglecta* were highly associated (Table 4), suggesting that accessions resistant to race TTKSK are likely to be resistant to races TRTTF and TTTTF. Association for the reaction to races TTSKS, TRTTF, and TTTTF was also observed in *Ae. triuncialis* (Table 4). Resistance with race specificity was observed in accessions of the remaining species, most noticeably in *Ae. cylindrica*, where only one accession exhibited resistance to race TRTTF.

**Table 4:** Probability from contingency tables for association analysis of the reactions of accessions of *Aegilops* ssp. to races TTKSK, TRTTF, and TTTTF of *Puccinia graminis* f. sp. *tritici*.

	TTKSK vs. TTTTF		TTKSK vs. TRTTF		TRTTF vs. TTTTF	
			
	*P-*value	Association^a^	*P-*value	Association	*P-*value	Association
*Ae. biuncialis*	<0.001	Highly associated	0.648	Independent	0.989	Independent
*Ae. caudata*	0.608	Independent	<0.001	Highly associated	0.740	Independent
*Ae. comosa*	0.014	Associated	0.158	Independent	0.038	Associated
*Ae. cylindrica*	0.037	Associated	0.756	Independent	0.545	Independent
*Ae. geniculata*	<0.001	Highly associated	<0.001	Highly associated	<0.001	Highly associated
*Ae. neglecta*	<0.001	Highly associated	0.011	Associated	<0.001	Highly associated
*Ae. peregrina*	0.615	Independent	<0.001	Highly associated	0.944	Independent
*Ae. triuncialis*	<0.001	Highly associated	<0.001	Highly associated	<0.001	Highly associated
*Ae. umbellulata*	<0.001	Highly associated	0.629	Independent	0.277	Independent


*^*a*^Based on *P* < 0.05.*

A group of 408 accessions resistant to races TTKSK, TRTTF, and TTTTF, were evaluated against US races TPMKC, RKRQC, QTHJC, and QFCSC. Three hundred ninety-six accessions remained resistant to all the races evaluated (Supplementary Table 1), indicating these accessions possess genes with broad spectrum resistance.

Sixty-five percent of the accessions evaluated in this study are native to Turkey or Greece. The frequencies of accessions resistant to all races from both countries were similar (30.6%) (Table 5). Higher frequencies of resistance were obtained in accessions from Macedonia (35.8%), France (38.9%), Iraq (46.7%), and Montenegro (75.0%), but the numbers of accessions evaluated from these countries were significantly smaller.

**Table 5:** Number and percentage of resistant *Aegilops* species accessions according to country of origin.

Country of origin	Number of accessions evaluated	Number of resistant accessions^1^	Percentage (%) resistant accessions
Turkey	624	191	30.6
Greece	307	94	30.6
Macedonia	53	19	35.8
Israel	49	7	14.3
Syria	42	9	21.4
Serbia	30	3	10.0
Cyprus	29	4	13.8
Ukraine	20	1	5.0
France	18	7	38.9
Azerbaijan	15	2	13.3
Iraq	15	7	46.7
Iran	13	2	15.4
Montenegro	12	9	75.0
Afghanistan	10	2	20.0
Others	67	15	22.4
Unknown	118	36	30.5


*^*1*^Accessions resistant against all *Pgt* races (TTKSK, TRTTF, and TTTTF) evaluated.*

Segregation ratios of the F_2_ progeny from biparental crosses between resistant and susceptible accessions indicated that resistance to race TTKSK in selected accessions is mostly conferred by single genes (Table 6). Eight resistant *Aegilops* accessions carry a single gene with dominant effect, whereas two resistant accessions carry a single gene with recessive effect. Two genes conferring resistance to race TTKSK were observed in three accessions of *Ae. triuncialis*. Inheritance with epistatic effect between two genes was also observed in four resistant parents. Segregation ratios of the F_2_ progeny of one *Ae. triuncialis* and one *Ae. umbellulata* resistant parent fit to a 9:7 ratio indicating the presence of a complementary gene action with duplicate recessive epistasis. Epistatic effect between two dominant genes was also observed in two *Ae. peregrina* resistant parents (Table 6), where the F_2_ progenies fit to a 11:5 ratio.

**Table 6:** Segregation of F_2_ populations of bi-parental crosses of *Aegilops* spp. to race TTKSK of *P. graminis* f. sp. *tritici*.

Species	Cross^a^	F_2_ plants				
		
		Resistant	Susceptible	Ratio tested (R:S)	X^2^	*P*-value
*Aegilops comosa*	PI 551049 (S) x PI 551054 (R)	28	108	1:3	1.412	0.235
*Aegilops cylindrica*	PI 554216 (S) x PI 254864 (R)	139	47	3:1	0.007	0.933
*Aegilops cylindrica*	PI 554216 (S) x PI 374345 (R)	140	32	3:1	3.752	0.053
*Aegilops cylindrica*	PI 554216 (S) x PI 568161 (R)	109	37	3:1	0.009	0.924
*Aegilops cylindrica*	PI 554216 (S) x PI 573369 (R)	104	27	3:1	1.346	0.246
*Aegilops peregrina*	PI 487274 (S) x PI 487278 (R)	24	69	1:3	0.032	0.858
*Aegilops peregrina*	PI 483010 (S) x PI 603931 (R)	107	55	11:5	0.550	0.458
*Aegilops peregrina*	PI 483010 (S) x PI 604185 (R)	127	57	11:5	0.002	0.937
*Aegilops peregrina*	PI 483010 (S) x PI 604193 (R)	135	49	3:1	0.261	0.610
*Aegilops triuncialis*	PI 173615 (S) x PI 219868 (R)	110	35	3:1	0.057	0.811
*Aegilops triuncialis*	PI 173615 (S) x PI 221899 (R)	59	41	9:7	0.307	0.579
*Aegilops triuncialis*	PI 173615 (S) x PI 254860 (R)	82	7	15:1	0.396	0.529
*Aegilops triuncialis*	PI 330492 (S) x PI 254861 (R)	71	25	3:1	0.056	0.814
*Aegilops triuncialis*	PI 330492 (S) x PI 374357 (R)	175	15	15:1	0.877	0.349
*Aegilops triuncialis*	PI 173615 (S) x PI 491436 (R)	150	11	15:1	0.093	0.760
*Aegilops umbellulata*	PI 542369 (S) x PI 298905 (R)	147	53	3:1	0.240	0.624
*Aegilops umbellulata*	PI 554395 (S) x PI 542375 (R)	90	64	9 :7	0.301	0.584


*^*a*^Female parent/Male parent; (R) and (S) indicate the resistant and susceptible parent, respectively.*

## Discussion

Races of *P. graminis* f. sp. *tritici*, such as the Ug99 race group, TKTTF and others detected from the contemporary *Pgt* populations worldwide, are a serious threat to bread and durum wheat production worldwide because of their broad virulence to many cultivars and rapid geographic spread. The limited number of stem rust resistance genes effective against these virulent races requires the identification of new sources of resistance. Different *Aegilops* species have contributed several stem rust resistance genes effective against race TTKSK including *Sr32, 33, 45, 46, 47, 51, 53, SrTA10187* and *SrTA10171* (Friebe et al., 1996; Schneider et al., 2008; Liu et al., 2011a,b; Olson et al., 2013a,b). However, only one gene, *Sr53*, is derived from *Ae. geniculata* in the tertiary genepool. Results from this study demonstrated that *Aegilops* species in the tertiary genepool of wheat are a rich source of resistance to race TTKSK and other *Pgt* races with broad virulence.

Although the overall frequency of resistant accessions in the entire *Aegilops* collection evaluated against races TTKSK, TRTTF, and TTTTF in this study was over 60%, we observed significant variation among species. Only two species (*Ae. geniculata* and *Ae. neglecta*) exhibited a high frequency of resistance (over 80%) against the three races. Interestingly in *Ae. biuncialis*, a species that also shares the same genome constitution as *Ae. geniculata* and *Ae*. *neglecta* (UUMM), the frequencies of resistance varied, exhibiting a high level of race specificity. Differences in the frequencies of resistance to stem, stripe, and leaf rust in species carrying the same genome have been also reported in the Section Sitopsis (SS genome) of *Aegilops* (Anikster et al., 2005; Scott et al., 2014). In species such as *Ae. geniculata* and *Ae. neglecta* where there is a high degree of association of the reactions to races TTKSK, TRTTF, and TTTTF, it is highly likely that the genes that confer resistance to one race is also effective against the other races. The progeny populations via bi-parental crosses initiated through this study will be further developed and analyzed to understand the genetic relationships for resistance to different races in these selected accessions.

Race specificity was a common feature observed in this *Aegilops* collection, as five species exhibited a percentage of accessions resistant to all three races TTKSK, TRTTF, and TTTTF below 20% (Table 3), and have no association of the reaction of two out of three races. Previous studies also report race specificity in *Aegilops* species (Olivera et al., 2007; Scott et al., 2014). Since gene introgression from *Aegilops* species in the tertiary genepool is a long and laborious process, it is preferable to use accessions that carry stem rust resistance that is effective against multiple races. About 30% (396 accessions) were resistant against all the races evaluated, indicating the availability of potential sources of new and diverse stem rust resistance genes that could be very useful in wheat breeding programs. Most of these resistant accessions (84%) were from the tetraploid species *Ae. geniculata, Ae. neglecta*, and *Ae. triuncialis*. Additional studies are required to assess the diversity in these resistant accessions to allow the identification of donor accessions that are likely to contribute non-redundant stem rust resistance genes. Choosing resistant accessions from geographically diverse countries of origin and exhibiting different infection types for gene introgression is a first step to maximize the chances of capturing new and unique resistance genes (Anikster et al., 2005).

Sixty-five percent of the accessions evaluated in this study originated from Turkey or Greece, two countries having the largest numbers of *Aegilops* species. Turkey is known to be the center of diversity for *Aegilop*s (Eig, 1929), and 17 out of the 23 *Aegilops* species have been identified in its territory (van Slageren, 1994). The nine species evaluated in this study are present in Turkey. The number and frequency of resistant accessions from Turkey and Greece (*n* = 285, 30.6%) from this study demonstrated that valuable sources of new genetic variation for stem rust resistance are present in these countries.

A prior knowledge on the inheritance of resistance in wild wheat relatives will facilitate alien gene introgression into wheat. We produced 17 biparental crosses to investigate the inheritance of TTKSK resistance. These populations will be further developed to map resistance genes and to develop closely linked markers within the wild species. Simple inheritance of stem rust resistance was found in most selected resistant accessions. Our result of a single dominant gene segregating in the *Ae. umbellulata* biparental F_2_ population from a cross between PI 542369 and PI 298905 was confirmed in an F_3_ population and mapped to chromosome 2U (Edae et al., 2016). A similar approach will be followed to characterize the resistance identified in this study. Two stem rust resistance genes were identified in three *Ae. triuncialis* resistant parents. Further studies are needed to characterize the effectiveness of each resistance gene. Multiple stem rust resistance genes with different resistance profile were reported in *Ae. sharonensis* (Olivera et al., 2008; Yu et al., 2017). A more complex inheritance of stem rust resistance with genes exhibiting epistatic effects was also observed in three *Aegilops* species. These results highlight the value of studying the genetics of stem rust resistance in the wild relative before attempting wide crosses for gene transferring.

*Aegilops* species in the tertiary genepool do not possess genome(s) homologous to the cultivated forms, and gene transfer through homologous recombination cannot be achieved with these species (Harlan and de Wet, 1971). Cytogenetic techniques such as irradiation and chemical treatments, production of synthetic amphiploids, use of gametocidal chromosomes, or *Ph1* gene mutants may be required for gene introgression into the cultivated forms (Friebe et al., 1996; Zaharieva and Monneveux, 2006). However, the introgression of alien chromatin to substitute for homoeologous chromosome segments has the potential of a simultaneous introduction of deleterious DNA that can affect agronomic and quality traits of wheat (Feuillet et al., 2008; Wulff and Moscou, 2014). New sequencing technologies, like Genotyping-By-Sequencing, have allowed the development of genetic linkage maps in wild relatives of wheat with non-previous available markers, and the identification of closely linked markers that can facilitate the gene transfer process by reducing the introgressed alien chromatin segment into elite materials (Edae et al., 2016, 2017). The sources of resistance identified from the tertiary genepool will also serve as targets for resistance gene cloning. Cloned genes and their delivery as transgenes in single or multiple resistance gene cassettes will completely resolve the linkage drag problem and ensure the effectiveness and durability of genes derived from more distant relatives of wheat (Wulff and Moscou, 2014). Today, new cloning techniques like mutational genomics (MutRenSeq) (Steuernagel et al., 2016) and association genetics with R gene enrichment sequencing (AgRenSeq) (Arora et al., 2018) allow a rapid and cheaper discovery and cloning of resistance genes. These technologies are opening new doors for fully exploiting the richness and diversity of wild relatives for wheat improvement.

## Author Contributions

PO and YJ were involved in the experimental design and manuscript preparation. PO performed the experiments and completed the data analysis. MR was involved in manuscript preparation and revision.

## Conflict of Interest Statement

The authors declare that the research was conducted in the absence of any commercial or financial relationships that could be construed as a potential conflict of interest.
