# Supplementary material for: Identification of New Sources of Resistance to Wheat Stem Rust in Aegilops spp. in the Tertiary Genepool of Wheat
Source: Front Plant Sci. 2018 Nov 22;9:1719. doi: 10.3389/fpls.2018.01719 (PMC6262079; doi:10.3389/fpls.2018.01719)
Supplement: Supplementary file 1 [file Table_1.docx]

**Supplemental table 1.** Infection types of *Aegilops* ssp. accessions resistant to races TTKSK, TRTTF, TTTTF, TPMKC, RKRQC, QTHJC, and QFCSC of *Puccinia graminis* f. sp. *tritici* at the seedling stage.

| **Species** | **ID number** | **Origin** | **TTKSK** | **TRTTF** | **TTTTF** | **TPMKC** | **RKRQC** | **QTHJC** | **QFCSC** |
| --- | --- | --- | --- | --- | --- | --- | --- | --- | --- |
|  |  |  | **(04KEN156/04)** | **(06YEM34-1)** | **(01MN84A-1-2)** | **(74MN1409)** | **(99KS76A-1)** | **(75ND717C)** | **(06ND76C)** |
| *Ae. biuncialis* | PI362339 | Macedonia | ;N^1^ | ;1- | ; | ; | ;N | ,1- | ;1- |
| *Ae. biuncialis* | PI483012 | Cyprus | 2 | 2- | 2-; | 2- | 2- | , | 2+ |
| *Ae. biuncialis* | PI483030 | Cyprus | 2+ | 2- | 2- | 2- | 2- | 2- | 2 |
| *Ae. biuncialis* | PI483033 | Cyprus | 2 | 2-; | 2- | 2- | 2- | 2-; | 2 |
| *Ae. biuncialis* | PI542165 | Turkey | 2- | ;2= | 2- | 2- | 2- | 2- | 22- |
| *Ae. biuncialis* | PI564173 | Turkey | 2 | 2= | 2- | 2-; | 2- | 2- | 2- |
| *Ae. biuncialis* | PI573339 | Turkey | 22- | 2- | ;2- | 2- | 2- | 2-; | 2 |
| *Ae. biuncialis* | PI573349 | Turkey | 1; | 2- | ;1 | ;1- | 2-; | ;1- | ; |
| *Ae. biuncialis* | PI431599 | Unknown | 2- | 2- | 2- | 22+ | Z | 2- | 1- |
| *Ae. biuncialis* | PI574465 | Unknown | 2 | 2- | 2- | 0 | 2- | 2- | 2- |
| *Ae. biuncialis* | PI550941 | Unknown | ;1- | 2- | ; | ;1- | ;1- | ;11- | 0; |
| *Ae. biuncialis* | PI550953 | Greece | ;1- | 2- | ;N1- | ;N1- | ; | ; | ;1- |
| *Ae. biuncialis* | PI550957 | Greece | ; | 2- | ; | ; | ;1- | ; | ; |
| *Ae. biuncialis* | PI550982 | Greece | 1; | 2- | 2+ | 2+3 / ; | ; | 2- | 2+ |
| *Ae. biuncialis* | PI554157 | Greece | ;1- | 2+ | ;1- / 2 | ;1- | 23 | 1- | ;1- |
| *Ae. biuncialis* | PI554161 | Greece | 2- | 2- | 2-; | ;2= | ;1- | ;2- | 2- |
| *Ae. biuncialis* | PI554163 | Turkey | 2+ | 2 | 2- | 2- | 2- | 2- | 2 |
| *Ae. biuncialis* | PI554164 | Turkey | ;2- | 2- | 2 | ;2- | 22+ | 2- | ;1- |
| *Ae. comosa* | PI551049 | Greece | 11+; | 1+1; | 0; | 1; | 0; | 0; | ; |
| *Ae. geniculata* | PI170193 | Turkey | 2- | 2- | ;1- | 2- | 2 | 22- | 2+ |
| *Ae. geniculata* | PI170195 | Turkey | 2-; | ;2- | ;1- | ;1- | ;2= | 2-; | 2 |
| *Ae. geniculata* | PI170210 | Turkey | 2- | 2- | ;1- | ;1- | 2- | 2- | 2 |
| *Ae. geniculata* | PI287737 | Spain | 2- | 2-; | ;2- | 2- | 2 | 2- | 2-N |
| *Ae. geniculata* | PI361880 | Romania | 2-; | ;1- | ;1- | ;1- | ;1- | ;1- | 1; |
| *Ae. geniculata* | PI374324 | Macedonia | 2- | ;1- | ; | ; | ; | ; | 2- |
| *Ae. geniculata* | PI374338 | Serbia | ;2- | ;2= | ; | 2- | ; | ;2= | 0; |
| *Ae. geniculata* | PI374339 | Montenegro | 2 | ;1- | ;1- | 2- | 2- | 2-; | 2+ |
| *Ae. geniculata* | PI374361 | Montenegro | 2 | 2- | 2-; | 2- | 2- | 2- | 2 |
| *Ae. geniculata* | PI374374 | Macedonia | 2- | 2- | 2-; | 2- | 2 | 2- | 2 |
| *Ae. geniculata* | PI388754 | Morocco | 2-; | ;1- | ; | ; | ;1- | ;2- | ;1- |
| *Ae. geniculata* | PI487221 | Syria | 2 | 2- | 2- | 2- | 2 | 2- | 2 |
| *Ae. geniculata* | PI487222 | Syria | 2 | 2-; | 2- | ;2- | 2- | 2- | 2 |
| *Ae. geniculata* | PI487223 | Syria | 2-; | ;1- | ;1- | ;1- | 2-; | 2- | 1-; |
| *Ae. geniculata* | PI487224 | Syria | 2- | ;2- | ;1- | ;N1- | 2 | 2- | 2-;N |
| *Ae. geniculata* | PI487225 | Syria | 2 | 2-; | 2- | 2- | 22+ | 2- | ;1- |
| *Ae. geniculata* | PI487226 | Syria | 2- | ;1- | 2- | ;N2= | 2 | 2- | 2 |
| *Ae. geniculata* | PI487227 | Syria | 2- | ;2- | ; | 2- | 2- | 2- | 2- |
| *Ae. geniculata* | PI487228 | Syria | 2- | ;2= | 2- | 2-; | 2 | 2- | 2 |
| *Ae. geniculata* | PI487229 | Syria | 2-2; | ;1- | ; | ; | 2-; | 2-; | 2 |
| *Ae. geniculata* | PI487283 | Jordan | 2+ | 2- | 2- | 22+ | 2 | 2 | 2 |
| *Ae. geniculata* | PI491426 | France | 2 | ;2- | 2- | 2- | 2- | ;2- | 2- |
| *Ae. geniculata* | PI491427 | France | 2- | ;2- | ; / 2- | 2- | 2 | 2- | 2- |
| *Ae. geniculata* | PI491428 | France | 2+ | ;2- | ;N | 2-; | 2 | 2- | 2- |
| *Ae. geniculata* | PI491430 | France | 22+ | 22+ | 22+ | 2 | 2+ | 2 | 2 |
| *Ae. geniculata* | PI491433 | France | 2-N | ;1 | ;1- | ;1 | 2- | 2-; | 2- |
| *Ae. geniculata* | PI524952 | France | 2 | ; | 2-; | ;2= | 2 | 2 | 2 |
| *Ae. geniculata* | PI524953 | Italy | 2- | 2- | 2- | 2- | 2 | 2 | 2-N |
| *Ae. geniculata* | PI542180 | Italy | 2 | 2 | 2- | 2 | 22+ | 2 | 2 |
| *Ae. geniculata* | PI542183 | Italy | 22+ | 2-; | 2- | 2-; | 2 | 2- | 2+ |
| *Ae. geniculata* | PI542184 | Turkey | 2 | 2- | 2 | 2- | 2 | 2- | 2- |
| *Ae. geniculata* | PI542185 | Turkey | 2- | 22- | 2- | 2- | 2 | 2-; | 2 |
| *Ae. geniculata* | PI542186 | Turkey | 2- | 2- | 2- | 22+ | 2 | 2-; | 2 |
| *Ae. geniculata* | PI542187 | Turkey | 22- | 2-; | 2-; | 2- | 22+ | 2-; | 2- |
| *Ae. geniculata* | PI542188 | Turkey | 22- | 2-; | 2- | 2-; | 2- | 2-; | 2- |
| *Ae. geniculata* | PI542190 | Turkey | 2-; | 2- | 2- | 2- | 2- | ;2- | ;2- |
| *Ae. geniculata* | PI551083 | Turkey | 2 | 2-; | 2- | 2-; | 2+ | ;2- | 22+ |
| *Ae. geniculata* | PI551084 | Turkey | 2 | 2-; | 2- | 2- | 2+ | 2+ | 22+ |
| *Ae. geniculata* | PI551085 | Greece | 2 | 2-; | 2- | 2- | 2- | 2- | 2- |
| *Ae. geniculata* | PI551086 | Greece | 2-; | ; | ;2- | 2-; | 2-; | 2-; | 2- |
| *Ae. geniculata* | PI551088 | Greece | 2 | ;1- | 2- | 2- | 22+ | 2-; | 2+ |
| *Ae. geniculata* | PI551091 | Greece | 2 | ;1- | 2- | 2- | 2 | 2- | ;1- |
| *Ae. geniculata* | PI551092 | Greece | 2-; | 2- | 2- | 2- | 2 | 2 | 2 |
| *Ae. geniculata* | PI551093 | Greece | 2 | 2-; | ;1- / 2- | ;2- | ; | 2 | ;2- |
| *Ae. geniculata* | PI551094 | Greece | 22+ | ; | ;1- | ; | ;1- | 2-; | 1 |
| *Ae. geniculata* | PI551097 | Greece | 2-; | ; | ;1- | ;2- | 2- | 2 | 2 |
| *Ae. geniculata* | PI551098 | Greece | ;2- | ; | 2= | ; | 2-; | 2- | 2- |
| *Ae. geniculata* | PI551099 | Greece | 2-N | ;1- | ;2- | ;2- | 2-; | 2-; | 2-; |
| *Ae. geniculata* | PI551100 | Unknown | 2- | ; | ;1- | ;1 | 2- | 2- | 2- |
| *Ae. geniculata* | PI551101 | Greece | ; | ; | ; | ; | ; | ;1- | ;1- |
| *Ae. geniculata* | PI551102 | Unknown | 2 | 2- | 2- | ;2- | 2+ | ;1- | ;N1- |
| *Ae. geniculata* | PI551103 | Greece | 2-; | 2- | 2- | 2- | 2 | 2- | 2 |
| *Ae. geniculata* | PI551104 | Greece | 2 | ;1- | ;2- | 2- | 2- | 2- | 2- |
| *Ae. geniculata* | PI551105 | Greece | 2-; | ;2- | ; | ;2- | ;2- | ;1- | ;1- |
| *Ae. geniculata* | PI551107 | Greece | 2-; | ;1- | ;2= | ; | 2- | ;2- | 2- |
| *Ae. geniculata* | PI551108 | Greece | 2- / 2 | 22+ | 2- | 2- | 22+ | 2- | 2- |
| *Ae. geniculata* | PI551109 | Greece | 2-N | ;2- | ;N | ; | 2- | 2- | ;1- |
| *Ae. geniculata* | PI551111 | Greece | 2- | 2- | 2- | 22- | 2- | 2-; | 2- |
| *Ae. geniculata* | PI551112 | Greece | ;1- | ; | ; | ;1- | 1-; | ;1- | 2-; |
| *Ae. geniculata* | PI551114 | Greece | ;12= | ; | ; | ; | ;1- | ;2- | ;11- |
| *Ae. geniculata* | PI551115 | Greece | 2- | ; | ;12- | ;2- | ;2- | 2- | 2- |
| *Ae. geniculata* | PI551117 | Greece | 2-; | ;1- | ;2- | 2- | 2 | 2- | 2- |
| *Ae. geniculata* | PI551118 | Greece | 2 | 2- | 2- | 2- | 2 | 2- | 2- |
| *Ae. geniculata* | PI554278 | Greece | 2 | 2- | ;2- | 2- | ;2- | 2- | 2- |
| *Ae. geniculata* | PI554280 | Greece | 2- | 2-; | 2-; | 2- | ;2- | 2-; | ;2- |
| *Ae. geniculata* | PI554281 | Greece | 2- | 2- | 2-; | 2-2 | 2 | ;2- | ; |
| *Ae. geniculata* | PI554284 | Greece | 2+ | ;2= | 2- | 2- | 22+ | ;2- | ;2- |
| *Ae. geniculata* | PI554290 | Turkey | 2-; | ; | 2=; | ; | ; | ;1- | 2- |
| *Ae. geniculata* | PI564186 | Turkey | 2- | ;2= | ;2= | 2- | 2-; | ;2- | 2- |
| *Ae. geniculata* | PI574475 | Turkey | ; | 0; | 0; | ; | 0; | 0; | 0; |
| *Ae. geniculata* | PI614625 | Turkey | 2-; | ; | ;1- | ;1- | ; | ;2- | 1-; |
| *Ae. geniculata* | CIae43 | Croatia | ;12+ | ; | ;1- | ;11+ | ;1 | ;1- | 11+; |
| *Ae. geniculata* | CIae53 | Turkey | 2- | 0; | ;1- | ; | 2-; | ;1- | 2- |
| *Ae. geniculata* | CIae63 | Bulgaria | 2 | ;2= | ;2= | ;N | 2- | 2-; | 2+ |
| *Ae. geniculata* | CIae65 | Ukraine | 2+; | ; | ;1- | ;1 | ;1- | ; | 22+ |
| *Ae. geniculata* | PI276978 | Unknown | 22+; | ; | ;11- | ;1 | 1-; | ;1- | ;11- |
| *Ae. geniculata* | PI289578 | Unknown | 2-2 | ; | ;1- | ;1 | ;1- | ;1- | ;2- |
| *Ae. geniculata* | PI298899 | Unknown | ; | 2-; | 2 | ; | 1- | ; | ; |
| *Ae. geniculata* | PI330487 | Unknown | 2 | ; | ;1- | ;1- | 1-1; | ;11- | ;2- |
| *Ae. geniculata* | PI369574 | Unknown | 2+ | 2 | 2-; | 2- | 2- | 2-; | 2+ |
| *Ae. geniculata* | PI369575 | Unknown | 2+ | 2- | 2- | 2- | 2- | 2- | 2+ |
| *Ae. geniculata* | PI369576 | Turkey | 2+ | 2 | 2- | 2- | 2- | 2- | 2+ |
| *Ae. geniculata* | PI369577 | Unknown | ;1 | ;1 | ; | ;N | ; | 2- | ;1- |
| *Ae. geniculata* | PI369578 | Unknown | 2- | 2- | ;1- | 2- | ;1- | 22- | 22+ |
| *Ae. geniculata* | PI369579 | Unknown | 2- | ; | ;1- | ; | ; | 2- | ; |
| *Ae. geniculata* | PI369580 | Unknown | 22+; | ;1 | ;1- | ;11+ | ;1- | ;1- | ;11- |
| *Ae. geniculata* | PI374337 | Unknown | 2+ | ;1 | 2 | 2- | 2 | 2- | 2 |
| *Ae. geniculata* | PI524955 | Unknown | ; | ;2- | ; | ; | ; | 0; | 0; |
| *Ae. geniculata* | PI542182 | Unknown | 2+ | 2 | 2- | 2- | 2- | 22- | 2 |
| *Ae. geniculata* | PI560741 | Unknown | 2+ | 2 | 2- | 2- | 2+ | ;2- | 2 |
| *Ae. geniculata* | PI564189 | Montenegro | 2+ | 2-; | 2- | 2-; | 2 | 2- | 2- |
| *Ae. geniculata* | PI564191 | France | 2 | 2- | 2- | 2 | 2 | 2-2 | 2 |
| *Ae. geniculata* | PI564192 | Italy | 2-; | ; | ;2- | ; | ; | 2- | 2-; |
| *Ae. geniculata* | PI564193 | Turkey | 2 | 2- | 2- | 2- | 2- | ;2- | ;1- |
| *Ae. geniculata* | PI573371 | Turkey | 22+ | ; | ; | ;2- | ; | ; | ; |
| *Ae. geniculata* | PI573373 | Turkey | 2 | 2 | 2- | 2- | ;2- | ;2- | ;2- |
| *Ae. geniculata* | PI573374 | Turkey | 2+ | 2-; | 2-; | 2- | 2- | 2- | 2 |
| *Ae. geniculata* | PI573375 | Turkey | 2- | ;1- | ;2- | 2- | ;2- | ; | ; |
| *Ae. geniculata* | PI573376 | Turkey | 2- | ; | ; | ;1- | 2- | ;2- | ; |
| *Ae. geniculata* | PI573377 | Turkey | 2 | ; | 0 | ; | 0; | 0; | ; |
| *Ae. geniculata* | PI573378 | Turkey | 2 / ; | 0; | 0; | 2-; | 0; | ; | ; |
| *Ae. geniculata* | PI573381 | Turkey | ;2= | ; | 2-; | 2- | ; | ; | ;1- |
| *Ae. geniculata* | PI573382 | Turkey | 2 | ; | ;2= | ;2- | 0 | 0; | 0; |
| *Ae. geniculata* | PI573383 | Turkey | 2+ | ;2- | 2-2 | ;2- | ;2- | 0; | ; |
| *Ae. geniculata* | PI573384 | Turkey | ;1 | ;2= | 0 | ;2- | 0 | 0; | 0; |
| *Ae. geniculata* | PI573386 | Turkey | 0 | ;1- | 0; | ; | ; | ;1- | ;13 |
| *Ae. geniculata* | PI573387 | Turkey | 2-; | ; | ; | ; | ; | 0; | 0; |
| *Ae. geniculata* | PI573388 | Turkey | 2- | 0; | ;2- | ;1- | 2-; | ; | 0; |
| *Ae. geniculata* | PI573389 | Turkey | 2+ | ; | ;2= | ;2- | ;2- | ; | ; |
| *Ae. geniculata* | PI573392 | Turkey | 2- / 2 | ;1- | ;2- | ; | 2- | ; | ;1- |
| *Ae. geniculata* | PI573394 | Turkey | 2+ | 2-; | 2-; | ;1- | 2- | ;2= | ;1- |
| *Ae. geniculata* | PI573395 | Turkey | 2+ | ;1- | ;2- | 2- | 2- | 0; | ;2- |
| *Ae. geniculata* | PI573396 | Turkey | 2 | ;2= | ;1- | 2-; | 2 | 2-; | ;1- |
| *Ae. geniculata* | PI573397 | Turkey | 22+ | ;1- | ;1- | ;1- | 2- | 2-; | 2- |
| *Ae. geniculata* | PI573398 | Turkey | 2 | 2 | 2-; | 2- | ;2- | ;2- | ; |
| *Ae. geniculata* | PI573399 | Turkey | 2- | ; | ;2- | ; | ; | ; | ; |
| *Ae. geniculata* | PI573402 | Turkey | 2- | ;1 | ;1- | ; | ; | ; | ; |
| *Ae. geniculata* | PI573406 | Turkey | 2 | 2-; | 2-2 | 22- | ;2- | ;2- | 2-; |
| *Ae. geniculata* | PI573407 | Turkey | 2- | ; | 0; | 2- | 0; | ; | ; |
| *Ae. geniculata* | PI573408 | Turkey | 0; | 2- | ;1- | 2- | 0; | 0; | 0; |
| *Ae. geniculata* | PI573409 | Turkey | 2-; | ;2= | ;2- / ; | 2-; | 0; | ; | 1-; |
| *Ae. caudata* | PI263554 | Turkey | ;1- | ;1- | ; | ; | ; | ; | ; |
| *Ae. caudata* | PI298887 | Turkey | ; | ;2= | ;1- | ;1- | ; | ; | ; |
| *Ae. caudata* | PI298888 | Turkey | ; | ;2= | ;1- | ; | ; | ; | ; |
| *Ae. caudata* | PI298889 | Turkey | ; | 2=; | ; | ; | ; | ; | ; |
| *Ae. caudata* | PI369571 | Unknown | 0; | ; | ; | ;1- | 0; | ; | 0; |
| *Ae. caudata* | PI369572 | Unknown | ; | ;2- | ; | ; | 0; | ; | ; |
| *Ae. caudata* | PI369573 | Unknown | 0; | 2-; | ; | ; | ; | ; | 0; |
| *Ae. caudata* | PI542173 | Turkey | ; | ; | ; | ; | ; | ; | 0; |
| *Ae. caudata* | PI542205 | Turkey | ; / 2-; | 1; | ; | ;2- | ; | ; | ; |
| *Ae. caudata* | PI542206 | Turkey | ;N1- | 22- | ;1- | ;1- | ;1- | ;1- | ; |
| *Ae. caudata* | PI542207 | Turkey | ; | ;1- | ;1- | ; | ; | ; | ; |
| *Ae. caudata* | PI542210 | Turkey | ;N1- | 22+ | ; | ;1- | ;1- | ;1- | ;1- |
| *Ae. caudata* | PI551121 | Greece | ; | ;1- | ;1- | ; | ; | ; | ; |
| *Ae. caudata* | PI551125 | Greece | ;N | 2 | 1-; | ; | ; | ; | ; |
| *Ae. caudata* | PI551126 | Greece | ;N | ; | ;1- | ;1- | ; | ; | ; |
| *Ae. caudata* | PI551138 | Greece | ; | ;2- | ;1- | 0; | ; | ; | 0; |
| *Ae. caudata* | PI551139 | Greece | ; | ;1- | ;1- | ; | ; | ; | ; |
| *Ae. caudata* | PI551142 | Greece | ;1- | ;1- | ;1- | ;1- | 1; | ;1- | ;1-1 |
| *Ae. caudata* | PI551143 | Greece | ; | 1-; | ;1- | ; | 0; | ; | 0; |
| *Ae. caudata* | PI551144 | Greece | 0 | ;1- | 1-; | ; | ; | 0; | 0; |
| *Ae. caudata* | PI551147 | Greece | ; | 2- | ;1- | ; | ;1- | ; | ; |
| *Ae. caudata* | PI551148 | Greece | ; | ;1- | ;1- | 0; | ; | ; | ; |
| *Ae. caudata* | PI551149 | Greece | ; | ;1- | ;1- | ; | ; | ; | ; |
| *Ae. caudata* | PI551150 | Greece | ; | ;1- | 1- | ; | ; | ; | ; |
| *Ae. caudata* | PI564194 | Turkey | ; | ;1- | 1-; | ;1- | ;1- | ; | ; |
| *Ae. caudata* | PI564195 | Turkey | ; | ; | ;1- | ;1- | ; | ; | ;1- |
| *Ae. caudata* | PI573412 | Turkey | 0; | ;1- | ;1- | ;1- | 0; | 0; | ; |
| *Ae. caudata* | PI573415 | Turkey | ; | ; | ;1- | ;1- | ; | 0; | 0; |
| *Ae. neglecta* | CIae55 | Unknown | ; | ;1- | 1-; | ;1- | 1-; | ;2= | ;11- |
| *Ae. neglecta* | PI170198 | Turkey | 0; | ; | 2- | ; | 2- | ; | ; |
| *Ae. neglecta* | PI170199 | Turkey | 2- | 2-; | 2 | 1-1 | 2-; | ; | ; |
| *Ae. neglecta* | PI170200 | Turkey | ; | 2-; | 2-; | ;1- | 22- | ; | ; |
| *Ae. neglecta* | PI170205 | Turkey | ; | ; | 2- | ;1- | ;1- | ; | 0; |
| *Ae. neglecta* | PI170208 | Turkey | 2-; | 2=; | 2+ | ; | ;1- | ;1- | ; |
| *Ae. neglecta* | PI170209 | Turkey | ;1 | ;2= | 22+ | 2- | 2- | 0; | ;1- |
| *Ae. neglecta* | PI170214 | Turkey | 0; | ; | ;1- | ; | 0; | ; | ; |
| *Ae. neglecta* | PI170215 | Turkey | ; | ; | ; | 0; | 0; | 0; | 0; |
| *Ae. neglecta* | PI172356 | Turkey | ; | 2- | 2- | ;2- | 2 | ;2- | 2-; |
| *Ae. neglecta* | PI173616 | Turkey | 2-; | 2-; | 22+ | ; | 2- | ; | ;2- |
| *Ae. neglecta* | PI178821 | Turkey | ; | ; | ;1- | ; | ; | 0; | ; |
| *Ae. neglecta* | PI179160 | Turkey | ;2- | 1-; | 22+ | 0; | 2- | ;1- | ;1- |
| *Ae. neglecta* | PI179161 | Turkey | ; | ;1- | 1-1 | ; | ;1- | ;1- | ;1- |
| *Ae. neglecta* | PI263555 | Turkey | 2-; | 2- | 1 | 2- | 2- | 2- | ;1- |
| *Ae. neglecta* | PI266816 | Unknown | 2 | 2- | 2 | ;2- | 2 | 2+ | 2- |
| *Ae. neglecta* | PI267987 | Portugal | ;2- | 2- | 2- | 2- | 2- | ; | ; |
| *Ae. neglecta* | PI276986 | Unknown | 2 | 2- | 2- | 1; | 22+ | 2 | 2 |
| *Ae. neglecta* | PI276987 | Turkey | ; | ;1- | 2- | ;1- | 2- | ; | ;1- |
| *Ae. neglecta* | PI276989 | Unknown | 2+ | 2- | 2 | 1; | 2+ | 2 | 22+ |
| *Ae. neglecta* | PI298890 | Iraq | 2- | 2- | 2- | ;1 | 2 | 2 | ;1- |
| *Ae. neglecta* | PI298894 | Turkey | 2-; | 2- | 2- | ;1- | 2- | ;2- | ;1- |
| *Ae. neglecta* | PI298895 | Iraq | ; | 2-; | 2- | ;1 | 2- | ;2- | ; |
| *Ae. neglecta* | PI298896 | Iraq | 2-; | 2- | 2- | ;1- | 2- | ;1- | 1-; |
| *Ae. neglecta* | PI298897 | Iraq | ;2- | 2- | 2- | ;1 | ;2- | ;2- / 22+ | ; |
| *Ae. neglecta* | PI298898 | Iraq | 2- | 2- | 2- | ;1- | 2-; | 2- | ;1- |
| *Ae. neglecta* | PI321698 | Unknown | ; | 2-; | 2- | ;1- | 2 | ; | ; |
| *Ae. neglecta* | PI344776 | Macedonia | ; | ;1- | ;11- | ; | ;1 | ;1- | ;11- |
| *Ae. neglecta* | PI344780 | Macedonia | 2=; | 2-; | 2- | 2- | 2 | 2-; | 2-; |
| *Ae. neglecta* | PI344781 | Macedonia | ;2= | 2=; | 2 | 2- | 2 | 2-; | 2-; |
| *Ae. neglecta* | PI344782 | Macedonia | 2- | 2-; | 2 | 2-; | 2 | ;2- | 2-; |
| *Ae. neglecta* | PI344784 | Macedonia | 2= | 2- | 2- | 2- | 2 | 2-; | ;2- |
| *Ae. neglecta* | PI344791 | Macedonia | 2=; | 2-; | 2- | 2- | 2 | 2- | ;1- |
| *Ae. neglecta* | PI372463 | Greece | ; | 2-; | 2- | ;2- | 2-; | ; | ; |
| *Ae. neglecta* | PI374317 | Montenegro | 2-; | 2- | 2 | 2- | 2 | 2-; | ;2- |
| *Ae. neglecta* | PI374318 | Macedonia | 2-; | 2- | 2 | 2- | 2- | 2-; | ;2= |
| *Ae. neglecta* | PI374319 | Montenegro | 2-; | 2- | 2- | 2- | 2 | 2- | 2-; |
| *Ae. neglecta* | PI374330 | Macedonia | 2-; | 2- | 2 | 22- | 2- | 2- | 2-; |
| *Ae. neglecta* | PI374335 | Montenegro | 2- | 2- | 2 | 2- | 2- | 2-; | 2- |
| *Ae. neglecta* | PI374336 | Macedonia | 1-; | ;1- | 2-; | ; | 2- | ;1- | ; |
| *Ae. neglecta* | PI374360 | Macedonia | 2- | 2- | 2- | 2- | 2 | 2-; | ;2- |
| *Ae. neglecta* | PI374368 | Montenegro | 2-; | 2-; | 2-2 | 2- | 2- | 2- | ;2- |
| *Ae. neglecta* | PI374370 | Montenegro | 2- | 2-; | 2- | 2- | 2- | 2- | 2-; |
| *Ae. neglecta* | PI374371 | Montenegro | 2 | 2- | 22- | 2 | 2+ | 2 | 22+ |
| *Ae. neglecta* | PI374375 | Macedonia | 2- | 2- | 2- | 2- | 2- | 2 | 2- |
| *Ae. neglecta* | PI378191 | Serbia | 2-; | 2- | 2- | 2- | 2- | 2- | ; |
| *Ae. neglecta* | PI486253 | Turkey | ; | 2-; | 2- | ; | ;2- | 2-; | ; |
| *Ae. neglecta* | PI486255 | Turkey | ;1- | 2-; | 2 | 0; | 2- | 2- | 0; |
| *Ae. neglecta* | PI486259 | Turkey | 22+ | 2-; | ;1- | ;1- | 2+ | 2 | 2-; |
| *Ae. neglecta* | PI542212 | Turkey | 2 | 2- | 2 | ; | 22+ | 2 | ; |
| *Ae. neglecta* | PI542214 | Turkey | ;2- | 2-; | 2 | 2- | 2- | 2- | ;2- |
| *Ae. neglecta* | PI542216 | Turkey | 2+ | 2-; | 2- | 1; | 2 | ;2- | 2+ |
| *Ae. neglecta* | PI542225 | Turkey | ;1- | 0 | 0 | 2- | 1- | 0; | 0; |
| *Ae. neglecta* | PI542226 | Turkey | 2 | ;1- | 1-1 | 1; | 1; | 2- | 1 |
| *Ae. neglecta* | PI542227 | Turkey | 2= | 2- | 2+ | - | 2- | 2- | 2- |
| *Ae. neglecta* | PI542228 | Turkey | ;1- | 2- | 22+ | 2-; | 2- | ;1- | ;1- |
| *Ae. neglecta* | PI542229 | Turkey | 2-2; / 2+ | 2- | 2 | 2- | 22+ | 2 | ;2- |
| *Ae. neglecta* | PI542232 | Turkey | ; | ;2- | 22+ | ; | ; | ; | 0; |
| *Ae. neglecta* | PI542233 | Turkey | ; | 2- | 2 | 0; | 2- | ; | 0; |
| *Ae. neglecta* | PI542234 | Turkey | ; | 2- | 2 | 2- | 2- | ;2- | ; |
| *Ae. neglecta* | PI551151 | Greece | ;1- | 2- | 2- | 2- / ; | 2- | ;2- | ;1- |
| *Ae. neglecta* | PI551152 | Greece | ;1- | 2-; | 2- | ;2- | 2-; | ;1- | ;1- |
| *Ae. neglecta* | PI551153 | Greece | 2 | 2- | 2- | 2- / 3- | 2 | 2 | 2 |
| *Ae. neglecta* | PI551155 | Greece | ; | 2-; | 2- | ; | 2-; | 2- | ;2- |
| *Ae. neglecta* | PI551156 | Greece | ; | ; | 2- | ; | 11-; | 2- | 1-; |
| *Ae. neglecta* | PI551157 | Greece | ; | ;2- | 2- | ;2- | 1-; | ; | ;1- |
| *Ae. neglecta* | PI551158 | Greece | ; | ;2- | 2- | 2- / ; | 2- | ;2- | ;2- |
| *Ae. neglecta* | PI551161 | Greece | ;1- | ;2- | 2- | 2-; | 1-; | ;2- | ;1- |
| *Ae. neglecta* | PI551162 | Greece | 1- | 2-; | 2- | 2- | 2- | ; | ; |
| *Ae. neglecta* | PI551163 | Greece | ; | 2-; | 2- | 2- | 2- | 2- | ;1- |
| *Ae. neglecta* | PI551164 | Greece | ; | 2-; | 2- | ; / 2- | 2- | 2-; | ;2- |
| *Ae. neglecta* | PI551165 | Greece | ; | ; | 2- | 2- | 2- | 2-; | ; |
| *Ae. neglecta* | PI551166 | Greece | 2-; | 2-; | 2- | 2- | 2- | ; | ;1- |
| *Ae. neglecta* | PI551167 | Greece | ; | 2- | 2 | 2-; | 2-; | 2-; | ;1- |
| *Ae. neglecta* | PI551168 | Greece | ; | 2-; | 2-; | ; | 2- | ;2- | ;1- |
| *Ae. neglecta* | PI551169 | Greece | ; | ;2= | 2- / 2-; | ; | 2-; | ;1- | ; |
| *Ae. neglecta* | PI551170 | Greece | 2- | 2- | 2- | 2- | 2- | ;1- | ;1- |
| *Ae. neglecta* | PI551171 | Greece | ; | 2- | 2-2 | ;1- | 2- | ;1- | ; |
| *Ae. neglecta* | PI551172 | Greece | ; | 2-; | 2- | ;1- | ;2- | ;1- | ; |
| *Ae. neglecta* | PI551173 | Greece | 2- | 2- | 2- | 2-; | 2- | ;2= | ;2= |
| *Ae. neglecta* | PI551175 | Greece | 0 | 2-; | 2 | ; | ; | ; | 0 |
| *Ae. neglecta* | PI551176 | Greece | ; | 2-; | 2- | ; | ;1- | 2- | ;N |
| *Ae. neglecta* | PI551177 | Greece | ; | ;2- | 2- | ; | ;1- | ; | ; |
| *Ae. neglecta* | PI554238 | Turkey | 0; | 2-; | 2-; | - | ; | ; | ; |
| *Ae. neglecta* | PI554243 | Turkey | 0; | 0; | 2- | ;2- | 2- | ;1- | ; |
| *Ae. neglecta* | PI554244 | Turkey | ;1- | 2-; | 2- | 2- | 2- | ; | ; |
| *Ae. neglecta* | PI554245 | Turkey | 2- | 2- | ; | ;2- | 2- | 2- | ;2- |
| *Ae. neglecta* | PI554246 | Turkey | 2=; | 2- | 2- | 2- | 2- | 2- | ;2- |
| *Ae. neglecta* | PI554247 | Turkey | 2-; | 2- | 2- | 2- | 2- | 2- | ;1- |
| *Ae. neglecta* | PI554253 | Turkey | 2-; | 2- | 2 | 2- | ; | ; | ; |
| *Ae. neglecta* | PI554254 | Turkey | 2- | 2- | 2 | ;2- | 2 | 2- | 1-; |
| *Ae. neglecta* | PI554255 | Turkey | 2= | 2-2 | 2 | 2- | 2 | 2-; | ; |
| *Ae. neglecta* | PI554256 | Turkey | 2=; | 2- | 22+ | 2- | 2 | 2-; | ;1- |
| *Ae. neglecta* | PI554261 | Turkey | ;1- | 2-; | 0; | ; | ;2- | ;1- | ;1- |
| *Ae. neglecta* | PI554263 | Croatia | 2- | 2- | 2- | 2- | 2- | 2- | 11- |
| *Ae. neglecta* | PI554265 | Croatia | ;1- | 2- | 2- | 2- | 2- | 2-; | ;1- |
| *Ae. neglecta* | PI554272 | Former Yugoslavia | 2-; | 2-; | 0; | ; | 2- | 2- | ; |
| *Ae. neglecta* | PI554286 | Turkey | ;1- | 2-; | 2- | 2- | ;1- | ; | ; |
| *Ae. neglecta* | PI560524 | Turkey | 2- | 2- | 2- | ;2- | 2- | ; | ; |
| *Ae. neglecta* | PI560525 | Turkey | 0 | 2- | ;2= | 0; | ; | 0; | 0; |
| *Ae. neglecta* | PI560526 | Turkey | 2=; | 2- | 2-; | 0; | 2- | ; | 0; |
| *Ae. neglecta* | PI560743 | Turkey | 2=; | 2-; | 2- | 2-; | 2- | ; | ; |
| *Ae. neglecta* | PI560746 | Turkey | 2- | 2-; | 2- | 2- | 2- | ; | ; |
| *Ae. neglecta* | PI564200 | Turkey | 0; | ; | 2-; | ; | ;1- | ; | ; |
| *Ae. neglecta* | PI564201 | Turkey | 0; | ;1- | 2- | ;1- | ;1- | ; | ; |
| *Ae. neglecta* | PI564202 | Turkey | 0; | 2-; | 2- | ; | 2- | ; | ; |
| *Ae. neglecta* | PI564203 | Turkey | ; | 2-; | ;2- | 2- | 2- | 2- | ; |
| *Ae. neglecta* | PI564204 | Turkey | 2- | 2-; | 2- | 11+ | ;1- | ;1- | 11-; |
| *Ae. neglecta* | PI564205 | Turkey | 2-; | 1-; | 2- | ;1- | ; | ;2- | 11-; |
| *Ae. neglecta* | PI564208 | Turkey | ; | ;2- | 2-; | ;2- | ; | ; | ; |
| *Ae. neglecta* | PI564209 | Turkey | 0; | ;2= | ; | ;2- | ; | ; | 0; |
| *Ae. neglecta* | PI564210 | Turkey | ; | 11-; | ; | ;1 | ; | ; | ; |
| *Ae. neglecta* | PI564211 | Turkey | 0; | 1-; | ;1- | ;1- | 0; | ; | 0; |
| *Ae. neglecta* | PI564212 | Turkey | ; | ;1- | ;1- | ; | ;1- | ;2- | 0; |
| *Ae. neglecta* | PI564213 | Turkey | ;1- | 2- | 2 | 2- | 22- | 22+ | 1-; |
| *Ae. neglecta* | PI564214 | Turkey | 2- | ;1- | ;2- | 2- | 2- | ;2- | 2- |
| *Ae. neglecta* | PI564215 | Turkey | ; | 2- | 2- | ;1- | ; | ; | ; |
| *Ae. neglecta* | PI564218 | Turkey | ; | ;2- | 2 | ;1- | ;2= | 0; | 0; |
| *Ae. neglecta* | PI573422 | Turkey | ;1- | ; | 2- / ;1- | ;1- | ; | 0; | 0; |
| *Ae. neglecta* | PI573423 | Turkey | 0; | ;2= | 2- | ; | 0; | 0 | 0; |
| *Ae. neglecta* | PI573424 | Turkey | ; | 22+ | 0; | ; | 2- | ; | 0; |
| *Ae. neglecta* | PI573425 | Turkey | ; | ;2= | 2-; / 2- | ; | ;1- | ;1- | ;1- |
| *Ae. neglecta* | PI573426 | Turkey | 2-; | ;2= | 2- | ;1- | 2- | 1;3 | 1-; |
| *Ae. neglecta* | PI573427 | Turkey | ; | ; | ; | ; | ; | ; | ; |
| *Ae. neglecta* | PI573428 | Turkey | ; | ; | ;1- | ; | ; | ; | ; |
| *Ae. neglecta* | PI573429 | Turkey | 1-; | ;2- | 0; | ; | ;1- | ; | 1-; |
| *Ae. neglecta* | PI573430 | Turkey | ;N | 0; | ;2- | ; | ; | ; | 0; |
| *Ae. neglecta* | PI573431 | Turkey | ;1- | ;1- | ; | ; | ; | ; | ; |
| *Ae. neglecta* | PI573432 | Turkey | ; | ;2= | ;2- | 2- | 2 | 1;3 | ; |
| *Ae. neglecta* | PI573433 | Turkey | ; | ;1- | ; / 2 | 2-; | ;1- | 0; | ; |
| *Ae. neglecta* | PI573434 | Turkey | 2= | 2-; | ;1- | ; | ;1- | ;2- | ;1- |
| *Ae. neglecta* | PI573435 | Turkey | 2= | 2-; | 2- | 2- | 2-; | ;1- | ; |
| *Ae. neglecta* | PI573437 | Turkey | ;2= | 1- | 0; | ;1- | ;1- | ; | 0; |
| *Ae. neglecta* | PI573440 | Turkey | 2- | 2- | 2- | ;2= / 2 | 2- | 2- | ; |
| *Ae. neglecta* | PI573441 | Turkey | 1-; | ; | 2- | ;1- | ;1- | ; | ; |
| *Ae. neglecta* | PI573442 | Turkey | ; | ;1- | ;N1- | 2- | 2-; | ; | 0; |
| *Ae. neglecta* | PI573443 | Turkey | ; | ; | 1 | 2- | ; | ; | 0; |
| *Ae. neglecta* | PI573444 | Turkey | 2- | 2- | 2- | 2-; | ; | ;1- | 0; |
| *Ae. neglecta* | PI573445 | Turkey | 2- | 2-; | 2- | 2- | 2- | ; | ; |
| *Ae. neglecta* | PI573446 | Turkey | 2- | 2- | 2- | 2- | ;2- | ;2= | ;2= |
| *Ae. neglecta* | PI573447 | Turkey | 2-; | 2- | 2- | 2- | 2- | ;2= | ; |
| *Ae. peregrina* | PI604173 | Israel | ; | 22- | 22+ | 1; | ; | ; | 2+ |
| *Ae. peregrina* | PI604174 | Israel | ; | 2- | 0; / 2- | ; | ; | ; | 2+ |
| *Ae. peregrina* | PI604181 | Israel | 0; | ; | ;1- | 0; | ; | 0; | ;1- |
| *Ae. peregrina* | PI604182 | Israel | 0; | 0; | 0; / X- | ; | ; | 0; | ; |
| *Ae. peregrina* | PI604186 | Israel | ; | ; | ;1- | ; / 2 | ;1- | 0; | 2- |
| *Ae. peregrina* | PI604193 | Israel | ; | 2-; | ; | 0; | 0; | 0; | ; |
| *Ae. triuncialis* | PI171468 | Turkey | ; | ; | ; | ; | ; | ; | ; |
| *Ae. triuncialis* | PI172684 | Turkey | 2- / 2 | 2- | 2 | ;N | 2-; / 3 | ;1- | ; |
| *Ae. triuncialis* | PI178820 | Turkey | ; | 2+ | ;1- | ;1- | ; | ;1- | ; |
| *Ae. triuncialis* | PI180793 | Turkey | 2+ | 22+ | 22- | 2-; | 2- | ;1- | 1-; |
| *Ae. triuncialis* | PI215781 | Afghanistan | ; | 11+ | ;1- | ;1- | ; | ; | ; |
| *Ae. triuncialis* | PI219868 | Unknown | 2- | 2- | 2-2 | 2 | 2- | 2 | 2-; |
| *Ae. triuncialis* | PI220330 | Iraq | ; | 2+ | ;2- | ;2- | ; | ; | ; |
| *Ae. triuncialis* | PI227340 | Unknown | ; | 1+1 | ;1- | ;1- | ; | ;1- | ; |
| *Ae. triuncialis* | PI250696 | Unknown | ; | 2 | ;1- | ;1- | ;1- | ; | ;1- |
| *Ae. triuncialis* | PI428559 | Iran | ; | 2- | 2- | - | 2- | ;2- | ;1- |
| *Ae. triuncialis* | PI428567 | Azerbaijan | 2-; / 2 | 2- | ;2- | 2- | ;2- | ;1- | ; |
| *Ae. triuncialis* | PI483038 | Azerbaijan | ; | 2- | ;1- | ;1- | ; | ; | ; |
| *Ae. triuncialis* | PI542330 | Cyprus | ; | 11+; | ;1- | ; | ; | ; | ;1- |
| *Ae. triuncialis* | PI551212 | Turkey | ; | 1 | ;1- | ; | ; | ; | ; |
| *Ae. triuncialis* | PI551217 | Greece | ; | 2 | ;1- | ;1- | ; | ;1- | ;1- |
| *Ae. triuncialis* | PI551218 | Greece | ; | 2 | ;1- | ; | ;1- | ; | ; |
| *Ae. triuncialis* | PI551220 | Greece | 2-; / 22+ | 2+ | 2+2 | 2 | 2 | 2-; | 2- |
| *Ae. triuncialis* | PI551222 | Greece | ; | 1 | ;1- | ;1 | 0; | ; | ; |
| *Ae. triuncialis* | PI568163 | Greece | ;1- | 2- | ;1- | ; | ; | ;2- | 2-; |
| *Ae. triuncialis* | PI573476 | Uzbekistan | ; | ;1 | ; | ; | 0; | ; | ; |
| *Ae. triuncialis* | PI170202 | Turkey | 2- | 2 | ;1- | ;1- | 2- | 2-13 | ;2= |
| *Ae. triuncialis* | PI170207 | Turkey | 2- | 2+ | 2-; | 2-; | 2- | 1;3 | 22+; |
| *Ae. triuncialis* | PI203437 | Turkey | ;1 | 22+ | ; | ;1- | ; | Z | 1-; |
| *Ae. triuncialis* | PI205277 | Turkey | 2-; | 2- | 2-2 | 2- | ; | ;2- | ; |
| *Ae. triuncialis* | PI263556 | Turkey | ; | 2 | ;1- | ;1- | ; | ; | ; |
| *Ae. triuncialis* | PI289581 | Turkey | ; | 11+ | ; | ;1- | ;1- | ;1- | ; |
| *Ae. triuncialis* | PI298903 | Unknown | ; | 2+ | ;1- / 2- | ;1- / 2- | ;1- / 2+ | ;1- | 1-; |
| *Ae. triuncialis* | PI344783 | Turkey | ;1- | 11+ | ;1- | ;1- | ; | ; | ;1- |
| *Ae. triuncialis* | PI344793 | Macedonia | ; | 11+; | ;1- | ;1- | ; | ; | ; |
| *Ae. triuncialis* | PI344795 | Macedonia | ; | 11+; | ;1- | ;1- | ; | ; | ;1- |
| *Ae. triuncialis* | PI361882 | Macedonia | ; | 11+; | ; | ; | ;1- | ;1- | ;1- |
| *Ae. triuncialis* | PI369629 | Unknown | 2+ | 2+ | 22-; | 2-; | 2 | ; | ; |
| *Ae. triuncialis* | PI369631 | Unknown | ; | 2+ | ;1- | ; | ; | ; | ; |
| *Ae. triuncialis* | PI369633 | Unknown | ; | 2 / 2+ | ; | ;1- | ; | ;1- | ;1- |
| *Ae. triuncialis* | PI369634 | Unknown | ; | 2+ | ;1- | ; | ; | ;1- | ; |
| *Ae. triuncialis* | PI369635 | Unknown | ; | 22+ | ; | ; | ; | 11-; | 1-; |
| *Ae. triuncialis* | PI369638 | Unknown | ;1- | 2+ | ;1- | ; | ; | ;1- | ; |
| *Ae. triuncialis* | PI369640 | Unknown | ; | 11+; | ;1- | ; | ;1- | ;1- | ;1- |
| *Ae. triuncialis* | PI369643 | Unknown | ;1- | 22+ | ;1- | ; | ; | ;1- | ; |
| *Ae. triuncialis* | PI369645 | Unknown | ; | 22+ | 1-; | ; | ; | ;1- | 1-; |
| *Ae. triuncialis* | PI374331 | Unknown | ; | 1;1+ | ; | ; | ;1- | ; | ;1- |
| *Ae. triuncialis* | PI374362 | Macedonia | ;1- | ;1 | ;1- | ; | ; | ; | ; |
| *Ae. triuncialis* | PI486282 | Macedonia | 2-; | 2- / 2+ | ;1- | ; | 2-; | 2-; | 1-; |
| *Ae. triuncialis* | PI486284 | Turkey | ; | 2+ | ;1- | ; | ; | ; | 1-; |
| *Ae. triuncialis* | PI486289 | Turkey | ; | 2- | 2-2 / ;1- | ; | 2- | ;1- | 1-; |
| *Ae. triuncialis* | PI486295 | Turkey | ; / 2- | 2 | ; | 2- | ; | 2- | ;2- |
| *Ae. triuncialis* | PI486296 | Turkey | 2-; | 2+2 | 2- | 2-; | 2- | 2 | ;2- |
| *Ae. triuncialis* | PI542286 | Turkey | ;1- | 2- | 2- | ; | ; | 2- | ;2- |
| *Ae. triuncialis* | PI542287 | Turkey | ;1- | 2- | ;1- | ;1- | ;2- | 2-; | ;2- |
| *Ae. triuncialis* | PI542297 | Turkey | 1 | 2 | ; | ; | ;1- | ;1- | 1- |
| *Ae. triuncialis* | PI542298 | Turkey | 1 | 2- | ;1- | ; | ;1-1 | ;1- | 1- |
| *Ae. triuncialis* | PI542327 | Turkey | ; | 2+ | ; | 0; | ; | 1-; | ;1- |
| *Ae. triuncialis* | PI542332 | Turkey | ; | 2+ | ; | ; | ; | ; | ;1- |
| *Ae. triuncialis* | PI542333 | Turkey | 2- | 2- | ;2- | ;1- | ;1- | ; | ; |
| *Ae. triuncialis* | PI542339 | Turkey | 2- | 2+ | ;1- | 2-; | ; | ;2- | 2-; |
| *Ae. triuncialis* | PI542344 | Turkey | ; | 2 | ;1- | ;2- | ; | ; | ;2- |
| *Ae. triuncialis* | PI542346 | Turkey | ;2= | 22+ | 2- | 2-; | 2-; | ; | ; |
| *Ae. triuncialis* | PI551181 | Turkey | ;1- | 11+ | ;1- | ; | ; | ; | ;1- |
| *Ae. triuncialis* | PI551196 | Greece | ; | 11+ | ;1- | ; | ; | ; | ; |
| *Ae. triuncialis* | PI551197 | Greece | ; | 11+; | ; | ; | ; | ; | ; |
| *Ae. triuncialis* | PI551203 | Greece | ; | 11+; | 0; | 0; | 0; | ; | 0; |
| *Ae. triuncialis* | PI551207 | Greece | ; | 11+ | ; | ; | ; | ; | ; |
| *Ae. triuncialis* | PI551208 | Greece | ; | 11+ | ;1- | ; | ;1- | ; | ; |
| *Ae. triuncialis* | PI551209 | Greece | ; | 2- | ; | ; | ; | ; | ;1- |
| *Ae. triuncialis* | PI551210 | Greece | ; | 2 | ;1- | ; | ; | ; | ; |
| *Ae. triuncialis* | PI551223 | Greece | ; | 2 | ;1- | ; | ; | ; | ; |
| *Ae. triuncialis* | PI551225 | Greece | ; | 11+; | ;1- | ; | ; | ; | ; |
| *Ae. triuncialis* | PI551238 | Greece | ; | 11+ | ;1- | ; | ;1- | ; | ; |
| *Ae. triuncialis* | PI551241 | Greece | ; | 2- | 1-; | ; | ; | ; | ; |
| *Ae. triuncialis* | PI551242 | Greece | ; | 2+ | ; | ; | ; | ;1- | ; |
| *Ae. triuncialis* | PI551244 | Greece | ; | 2 | ; | ; | ; | ;1- | ; |
| *Ae. triuncialis* | PI551245 | Greece | ; | 2 | ;1- | ; | ; | ;1- | ;1- |
| *Ae. triuncialis* | PI551246 | Greece | ; | 22+ | ;1- | ; | ; | ;1- | ; |
| *Ae. triuncialis* | PI551248 | Greece | ; | 2+ | ;1- | ; | ; | ; | ;1- |
| *Ae. triuncialis* | PI551249 | Greece | ; | 2- | ; | ; | ; | ; | ; |
| *Ae. umbellulata* | CIae66 | Serbia | ;2= | 2- | 22+ | ; | 2- | 2- | ;2- |
| *Ae. umbellulata* | PI542370 | Turkey | 2-N | 2- | 2- | 2- | 2- | 2+3 | 2+ |
| *Ae. umbellulata* | PI554282 | Turkey | 0; | ;1- | 1N | 2-; | 2-; | 0; | ; |
| *Ae. umbellulata* | PI554417 | Turkey | 2- | 2- | 2-; | 2-; | 2- | 2- | 2- |
| *Ae. umbellulata* | PI573516 | Turkey | 2- | 2 | 2-; | 2 | 22- | 2- | 2- |

^1^Infection types observed on seedlings at 14 days post-inoculation using a 0-4 scale according to Stakman et al. (1962), where ITs of ;, 1, 2, or X are considered as a low IT and ITs of 3 or higher are considered as a high IT. N denotes excessive necrosis, and Z denotes ordered distribution of variable-sized uredinia, larger uredinia towards the leaf tip. “/” indicated accessions were heterogeneous with predominant type given first. “-” indicated missing data.
